# From Trauma to Bonding: the Role of Attachment in the Relationship Between Child Sexual Abuse and Adult Intimacy

**DOI:** 10.1007/s40653-026-00845-y

**Published:** 2026-03-05

**Authors:** Vanesa Pastor-Cerezo, Alejandro Iborra Cuéllar

**Affiliations:** Department of Educational Sciences, University of Alcalá, Alcalá de Henares, Spain

**Keywords:** Child sexual abuse, Attachment, Intimacy, Self-disclosure, Relational trauma

## Abstract

Childhood sexual abuse (CSA) has been consistently associated with long-term difficulties in affective development, particularly in adult attachment and the capacity for intimacy. This study compared adult attachment patterns and intimacy levels between individuals with and without a self-reported history of CSA, using a sample of 587 Spanish-speaking adults, 161 of whom reported experiencing CSA. Participants completed validated measures of attachment and intimacy, and group differences were analyzed using MANOVAs. Multiple mediation analyses were conducted to explore whether attachment dimensions explained the link between CSA and intimacy outcomes, controlling for gender. Results showed that survivors of CSA exhibited significantly higher levels of attachment anxiety and avoidance, and lower socio-emotional competence. They also reported reduced intimacy and self-disclosure, particularly in relation to their personality and body. Mediation analyses indicated that attachment avoidance and low emotional competence mediated the relationship between CSA and all intimacy indicators. Interestingly, attachment anxiety was positively related to general intimacy but negatively associated with bodily self-disclosure. These findings highlight the indirect impact of CSA on adult intimacy through maladaptive attachment patterns. They suggest the need for trauma-informed interventions that go beyond memory processing to foster relational skills and embodied forms of safe intimacy—crucial elements for emotional healing in adulthood.

## Introduction

Child sexual abuse (CSA) is one of the most serious forms of maltreatment, and its effects can last well beyond childhood (Hébert et al., 2021). Various studies estimate that 10–20% of children have experienced some form of abusive sexual contact before the age of 18, with higher percentages for females than males (Pan et al., 2020; Piolanti et al., 2025; Wang, 2024), although it is recognized that these figures may be even higher due to silencing and low disclosure rates (Gemara & Katz, 2022; Landberg et al., 2022). CSA involves not only physical and emotional transgression, but also a breakdown in affective and relational development (MacIntosh & Ménard, 2021). These early experiences can affect how a person bonds with others throughout life, particularly with regard to the ability to form intimate relationships and to trust others emotionally (Ali et al., 2024).

From a developmental perspective, intimacy constitutes one of the central tasks of early adulthood. Following Erikson (1985), this stage implies the ability to share one’s inner world with another person without fear of losing one’s identity. For some people, especially those with traumatic childhood experiences, this process may be hindered by mistrust, fear of rejection or difficulty in showing vulnerability (Fitzgerald, 2021). Based on this approach, there are models that allow us to understand intimacy not as a dichotomous quality, but as a continuum in which different levels of adaptability can be identified (Orlofsky, 1976, 1993), so that they are understood as developmental positions within an affective continuum. For the purpose of this study, intimacy is conceived from a dual perspective: on the one hand, as the adaptive resolution of the developmental crisis of intimacy versus isolation (Erikson, 1985), where high values reflect the integration of positive attributes in relationships and the ability to establish meaningful ties, while low values are associated with the predominance of isolation (Orlofsky, 1976, 1993). On the other hand, intimacy is explored through the capacity for self-disclosure of intimate aspects (Choi & Toma, 2022), specifically regarding personality and body, with the understanding that greater openness in these spheres reflects a greater willingness to share the inner world and thus a greater capacity for intimacy (Poucher et al., 2022).

Attachment theory, meanwhile, provides a solid framework for understanding how early experiences influence the way people bond in adulthood. The attachment system, first described by Bowlby (1973), has as its primary function the search for security in situations of threat or distress, and is constructed through interaction with caregiving figures. When this bond is marked by neglect, abuse or affective inconsistency - as in cases of CSA - insecure attachment styles are more likely to develop (Noll, 2021), which persist throughout the life cycle and condition the person’s relational and emotional life (Fitzgerald, 2022).

In adult attachment (Melero & Cantero, 2024), attachment configurations are explained by the interaction of four dimensions: (1) anxiety determined by fear of abandonment, (2) avoidance defined as emotional distancing, (3) anger understood as hostility in the face of affective frustrations, and (4) socioemotional competence with which emotional management and relational skills are produced; and their combination allows us to identify different attachment styles. First, secure attachment involves socioemotional competence, favoring stable relationships (Sagone et al., 2023); second, fearful attachment combines anxiety and avoidance, generating ambivalence between desire for closeness and fear of exposure (Melero & Cantero, 2024). In contrast, preoccupied attachment presents anxiety and anger, fostering dependent and insecure relationships (Henschel et al., 2020); and, finally, distant attachment is characterised by avoidance, reflecting a preference for emotional self-sufficiency (Sagone et al., 2023). Studies show a higher prevalence of insecure attachment, especially fearful attachment, in people with a history of CSA (Huang et al., 2020; Rosa Midolo et al., 2020; Weetman et al., 2021), suggesting that early harmful experiences generate ambivalent attachments, where the desire for intimacy coexists with the fear of rejection.

While the impact of CSA is well-documented, there remains a significant research gap regarding how adult attachment —viewed from a dimensional perspective— interacts with the resolution of Erikson’s intimacy vs. isolation stage in survivors. Moreover, there is a lack of empirical evidence linking these constructs to specific domains of self-disclosure (both personality and body-related). This study addresses this gap by exploring how early trauma affects the multidimensional capacity for intimacy in adulthood. In this sense, the interrelationship between CSA, adult attachment and capacity for intimacy allows for an explanatory model in which early trauma disrupts key affective processes that enable a person to form deep and secure attachments. It is not just about the damage produced by the traumatic event, but how this is internalized and manifests, over time, in the way people bond (Riazi & Manouchehri, 2024; Van Assche et al., 2019). Understanding these connections is essential to advance knowledge about the sequelae of CSA and to guide interventions that support the development of healthy relationships in adulthood.

This is why the present study aims to compare adult attachment and intimacy in a sample of adults with and without a history of CSA, in order to identify significant differences between the two groups, trying to establish a differential profile that can contribute to the design of interventions that are more tailored to the specific needs of this population. Therefore, it is hypothesized that the group of CSA victims will present greater attachment difficulties, manifested in a higher prevalence of insecure attachment styles (such as fearful or preoccupied) and lower levels of intimacy (both in the resolution of the developmental crisis and in the self-disclosure of personal and bodily aspects), compared to the group without a history of CSA.

## Methodology

The present study was conducted using a quantitative methodology, following an ex post facto comparative design. The research focused on analyzing the dependent variables of adult attachment and intimacy in relation to the independent variable of the presence or absence of experiences of CSA. The online questionnaire was distributed through a variety of channels such as universities and associations that collaborated in the initial dissemination among their communities. Data collection was carried out between October 2024 and May 2025. The average time to complete the questionnaire was 15–20 min, which was included as additional information for participants in the questionnaire. Participation was voluntary and no financial compensation or incentives were provided.

The announcement was neutral and open to Spanish-speaking adults; no quotas or filters related to childhood sexual abuse (CSA) were established in the recruitment process. CSA status was determined exclusively within the questionnaire, using a retrospective item with a standardized definition. This method facilitated outreach to a diverse population and the inclusion of individuals who may have had experiences of CSA, given the sensitive nature of the research topic. Prior to the start of the questionnaire, all participants were informed of the data processing protocols, in strict compliance with the General Data Protection Regulation (EU) 2016/679, Organic Law 3/2018 and Organic Law 1/1982. Informed consent was a prerequisite question in the questionnaire; lack of consent resulted in immediate termination of the survey, thus ensuring voluntary and ethical participation. The research was approved by the Research Ethics Committee with code CEID2021/6/132.

### Research Participants

The sample consisted of Spanish-speaking adult (*N* = 587), recruited through a non-probability snowball sampling process. First, descriptive analyses were carried out for the socio-demographic variables of gender, age and level of education, in order to understand the characteristics of the sample. In terms of gender, 81.1% (*N* = 475) identified themselves as women, 18.4% (*N* = 108) as men, and 0.3% (*N* = 2) did not identify with any of the above. In addition, the distribution of the variables of educational level and age was observed (Table 1), with both variables having a missing value. For this last variable, coding was carried out, so that two groups with similar distributions were established to consider the developmental moment in which they were, so that (1) participants aged 24 years or less (*N* = 247), and (2) participants over 24 years (*N* = 338) were maintained.

**Table 1:** Distribution of the Sample by Educational Level and Age Variables

Variable	Categories	*N*	%
Education level	Compulsory	22	3,8
	Third level	211	36
	Post-compulsory university (Degree)	170	29
	Post-compulsory higher university (Master, PhD)	178	30,4
Age	Between 18 and 19	129	22
	Between 20 and 21	56	9,6
	Between 22 and 24	62	10,6
	More than 24	338	57,7

Regarding the variable of abuse, we explored how many participants identified themselves as victims (*N* = 161) and how many did not (*N* = 425), and of the victims their distribution by gender representing 89.4% (*N* = 143) women, 10% (*N* = 16) men and 0.6% (*N* = 1) people who do not identify with these genders, showing a significant result (χ^2^ (2) = 10.872, *p* = 0.004).

### Instrument

For this study, an online self-administered questionnaire was used for data collection, divided into different sections for each of the study variables. Firstly, socio-demographic information was collected including age, categorized into specific ranges to fit the period of early adulthood and to avoid typographical errors in data collection; gender; and level of education attained. Secondly, CSA victimization was determined by means of a dichotomous question (yes/no). CSA was defined according to the criteria of Finkelhor and Hotaling (1984) and Sosa and Capafons (1996), referring to sexual acts between an adult and a minor with significant age or maturational differences. Participants were able to identified as victims based on two complementary conditions: (1) a 5-year age difference when the child was 12 or younger, or a 10-year difference when the minor was between 13 and 16 years old; and/or (2) sexual acts occurring through coercive techniques (threat, force, or deception) or involving a clear position of power over the child. The dependent variables of adult attachment and intimacy were collected through different validated questionnaires, administered in Spanish, and total scores for attachment dimensions were calculated as the mean of the items for each factor, while intimacy and self-disclosure scores were obtained by summing the items of their respective scales.

#### Adult Attachment

Adult attachment was assessed using the Adult Attachment Questionnaire Revised (CAA-r) (Melero & Cantero, 2024). This multidimensional instrument measures attachment through four key factors: (1) Anxiety, which reflects fear of abandonment or of not being liked (α = 0.883); (2) Anger, which indicates the propensity for hostile reactions in the context of bonding (α = 0.723); (3) Avoidance, which refers to the tendency to maintain emotional distance and self-sufficiency (α = 0.799); and (4) Socioemotional Competence, which assesses the ability to manage one’s own and others’ emotions in order to establish healthy relationships (α = 0.747). This instrument was chosen over others that only measure anxiety and avoidance because it is believed that taking into account the dimensions of socio-emotional competence and anger makes the dynamics of emotional bonds more complex and better captures them. These dimensions can be assessed from their percentiles to determine attachment style: secure, fearful, preoccupied and distant. Taking into account both dimensions and styles – dimensional and categorical perspective – facilitates understanding of how emotional bonds are formed.

#### Intimacy

Intimacy was explored using two complementary instruments. First, the Intimacy subscale of The Modified Erikson Psychosocial Stage Inventory (MEPSI) (Leidy & Darling-Fisher, 1995) was used, which assesses adaptive resolution of the developmental crisis of Intimacy vs. Isolation (α = 0.785). High scores on this scale indicate a greater ability to form meaningful ties, whereas low scores are associated with a predominance of loneliness or relational isolation. Second, the Personality self-disclosure (α = 0.853) and Body self-disclosure (α = 0.890) subscales of The Jourard Self-Disclosure Questionnaire (JSDQ) (Jourard & Lasakow, 1958) were included. These scales measure the capacity for self-disclosure of deep aspects related to one’s own personality and body. High scores on these subscales reflect a greater willingness to openness and vulnerability, crucial elements for deep intimacy.

### Statistical Analysis

Data analysis was carried out using SPSS statistical software, version 29.0. Initially, a preliminary descriptive analysis of all study variables was carried out. For the categorical variables (gender, educational level, age and CSA victimization), frequencies and percentages were obtained. Correlation tests were also carried out using Chi-square (χ^2^) and Pearson’s r test. Besides, to examine the differences between the group of CSA victims and the group with no history of CSA, different comparative analyses were used. Differences in the continuous dimensions of attachment and in the continuous components of intimacy were assessed using a multivariate ANOVA (MANOVA), with the presence/absence of CSA as a factor, as well as a MANOVA to assess the interaction of CSA and age variables on the intimacy variables. Moreover, for the comparison of the distribution of attachment styles (Secure, Fearful, Dismissing and Preoccupied) between the group of victims and non-victims, K-means cluster analyses were performed, and a contingency table was constructed, applying the Chi-square test (χ^2)^. Finally, mediation analyses were conducted using the PROCESS macro for SPSS (model 4). It was examined whether attachment dimensions mediated the relationship between the experience of CSA (yes/no) and each of the components of intimacy: developmental resolution of intimacy, self-disclosure about personality and self-disclosure about the body. All models included bootstrapping with 5000 samples to estimate 95% confidence intervals for indirect effects.

#### Treatment of Missing Data

Since there were missing values in the database, a protocol for their treatment was carried out. Initially, a listwise (Graham, 2009) was performed, excluding 1 case with a significant amount of missing data (> 50%). Once the case was removed, and with a minimum percentage of missing values in all variables (> 4%), the MCAR Little test was performed in order to explore whether there were patterns dependent on other variables, so that we could examine whether the distribution of the data was completely random (χ^2^ (834) = 818.075, *p* = 0.647). Given the test result, the data were shown to be completely random, which allowed the application of multiple imputation (Salgado et al., 2016), only for ordinal variables, since the application of an imputation on categorical variables, such as those that are sociodemographic, may imply a relevant bias in the research. Specifically, linear regression was used as a multiple imputation method, generating 5 complete data sets. The analysis of the descriptive statistics of the imputations performed revealed that one of the imputation models was an optimal fit to the data (Salgado et al., 2016), being the one selected for the subsequent analyses.

## Results

As a first step, descriptive analyses and correlation tests between the dependent variables were performed (Table 2), after the assessment and elimination of outliers from the sample: eight univariate outliers (SD > 3), and one multivariate outlier (the rest of the Mahalanobis distances showed a p-value > 0.001) were identified. Pearson correlation results showed that the attachment Socioemotional competence variable correlates positively with the intimacy variables, while the anxiety, avoidance and anger variables correlate negatively.

**Table 2:** Dependent Variables Correlations

	AAnx	ASC	AAv	AAn	I	SDP	SDB
AAnx	1						
ASC	− 0.469**	1					
AAv	0.475**	− 0.507**	1				
AAn	0.227**	− 0.094**	0.214**	1			
I	− 0.394**	0.712**	− 0.706**	− 0.199**	1		
SDP	− 0.392**	0.539**	− 0.492**	− 0.184**	0.560**	1	
SDB	− 0.376**	0.464**	− 0.412**	− 0.176**	0.455**	0.694**	1

Note. AAnx: attachment anxiety; ASC: attachment Socioemotional competence; AAv: attachment avoidance; AAn: attachment anger; I: intimacy; SDP: self-disclosure personality; SDB: self-disclosure body

Statistical note. **p < 0.01; *N* = 576

Given that resolution of the intimacy crisis is expected in early adulthood, it was explored differences for these variables across age groups through a MANOVA analysis, to study the possible interaction between the independent variables of CSA (two levels) and Age (two levels). The test results showed no interaction effects in the full model (Wilks’ Lambda = 0.991; F (3, 570) = 1.790, *p* < 0.001, ηp^2^= 0.009), although a significant effect does appear for the Intimacy variable (Table 3), but with a small effect size (F (1, 572) = 5.274, *p*= 0.022, ηp^2^ = 0.009).

**Table 3:** Means and Standard Deviations for Intimacy by Age and CSA Groups

CSA Group	24 years old or younger		More than 24 years old	
	No CSA (*N* = 204)	CSA (*N* = 42)	No CSA (*N* = 217)	CSA (*N* = 113)
Intimacy	3.832 (0.593)	3.773 (0.523)	3.985 (0.592)	3.635 (0.720)

To examine differences between the group of CSA victims and the group with no history of CSA, different comparative analyses were used. Differences in the continuous dimensions of attachment and in the continuous components of intimacy were assessed using a multivariate ANOVA (MANOVA) for each set of dependent variables (attachment on the one hand, intimacy on the other), with the presence/absence of CSA as a factor. Prior to the interpretation of each of these analyses, the assumption of homogeneity of the variance-covariance matrices was verified by means of Box’s M-test, coming out in both cases non-significant, so Wilks’ Lambda will be the primary multivariate criterion to determine the overall significance of both MANOVAs. First, the result of the MANOVA analysis for the attachment variables (Wilks’ Lambda = 0.852; F (4, 572) = 24.749, *p* < 0.001, ηp^2^= 0.148), showing a statistically significant result and a large effect size. Second, the MANOVA for the intimacy variables (Wilks’ Lambda = 0.958; F (3, 573) = 8.390, *p* < 0.001, ηp^2^= 0.042) also showed a significant result, although with a small effect size.

In both cases, inter-subject effects tests were conducted (Table 4), showing that, on the one hand, the group with a history of CSA victimization presented significantly higher means in Anxiety and Avoidance, with large and medium effect sizes, respectively. On the other hand, the group without a history of CSA victimization showed significantly higher means on Socioemotional Competence, Intimacy, Personality Self-Disclosure and Body Self-Disclosure, in all cases with small effect sizes. It should be noted that the attachment anger dimension did not show significant results.

**Table 4:** Dimensions of Attachment and Intimacy to CSA Victimization: Descriptive and Univariate Statistics

	Group	M (SD)	F	df	p	ηp^2^​
Anxiety	No CSA	3.269 (0.996)	88.527	1, 575	< 0.001	0.133
	CSA	4.151 (1.003)				
	Total	3.506 (1.071)				
Socioemotional competence	No CSA	4.455 (0.735)	16.817	1, 575	< 0.001	0.028
	CSA	4.171 (0.748)				
	Total	4.379 (0.748)				
Avoidance	No CSA	2.333 (0.846)	39.682	1, 247.743	< 0.001	0.072
	CSA	2.882 (0.956)				
	Total	2.480 (0.909)				
Anger	No CSA	2.779 (0.872)	3.399	1, 575	0.066	0.006
	CSA	2.931 (0.872)				
	Total	2.820 (0.877)				
Intimacy	No CSA	3.910 (0.596)	15.015	1, 247.881	< 0.001	0.028
	CSA	3.673 (0.673)				
	Total	3.847 (0.626)				
Self-disclosure for Personality	No CSA	34.464 (6.865)	17.803	1, 575	< 0.001	0.030
	CSA	31.687 (7.379)				
	Total	33.718 (7.108)				
Self-disclosure for Body	No CSA	36.016 (7.996)	18.316	1, 575	< 0.001	0.031
	CSA	32.771 (8.276)				
	Total	35.144 (8.192)				

N_total_ = 577; N_NoCSA_ = 422; N_CSA_ = 155

Note. For the Avoidance and Intimacy variables, significance was based on Welch’s test due to violation of the homogeneity of variances assumption

After this, to classify participants by attachment styles, a hierarchical cluster analysis was performed. Ward’s method and Euclidean squared distance produced four clusters, with a rescaled distance of four. Based on Melero and Cantero (2024), a k-means clustering procedure was subsequently performed using these clusters as non-random starting points. The frequencies within each attachment style were: Secure (31%, *N* = 179) in which high levels of Socioemotional Competence were shown, and low levels in the other dimensions; Preoccupied (22.5%, *N* = 130) with high levels in its predominant dimension, anxiety, and medium in the other dimensions; Dismissing (23.1%, *N* = 133) with high levels of avoidance and anger, and medium or medium-low in socioemotional competence and anxiety; and Fearful (23.4%, *N* = 135) with high levels in all dimensions, except for socioemotional competence.

Regarding the distribution of the victimization groups in the different attachment styles (Table 5), a significant result was shown in the Chi-square test (χ^2^ (3) = 61.897, *p* < 0.001). It shows that the predominant attachment style in the victim group is the Fearful attachment style, and in the non-victim group the Secure attachment style.

**Table 5:** Frequencies and Percentages for Attachment Styles by CSA Groups

		Secure	Dismissing	Preoccupied	Fearful
No CSA	%	36,7	26,8	19,9	16,6
	N	155	113	84	70
CSA	%	15,5	12,9	29,7	41, 9
	N	24	20	46	65

**Fig. 1:**
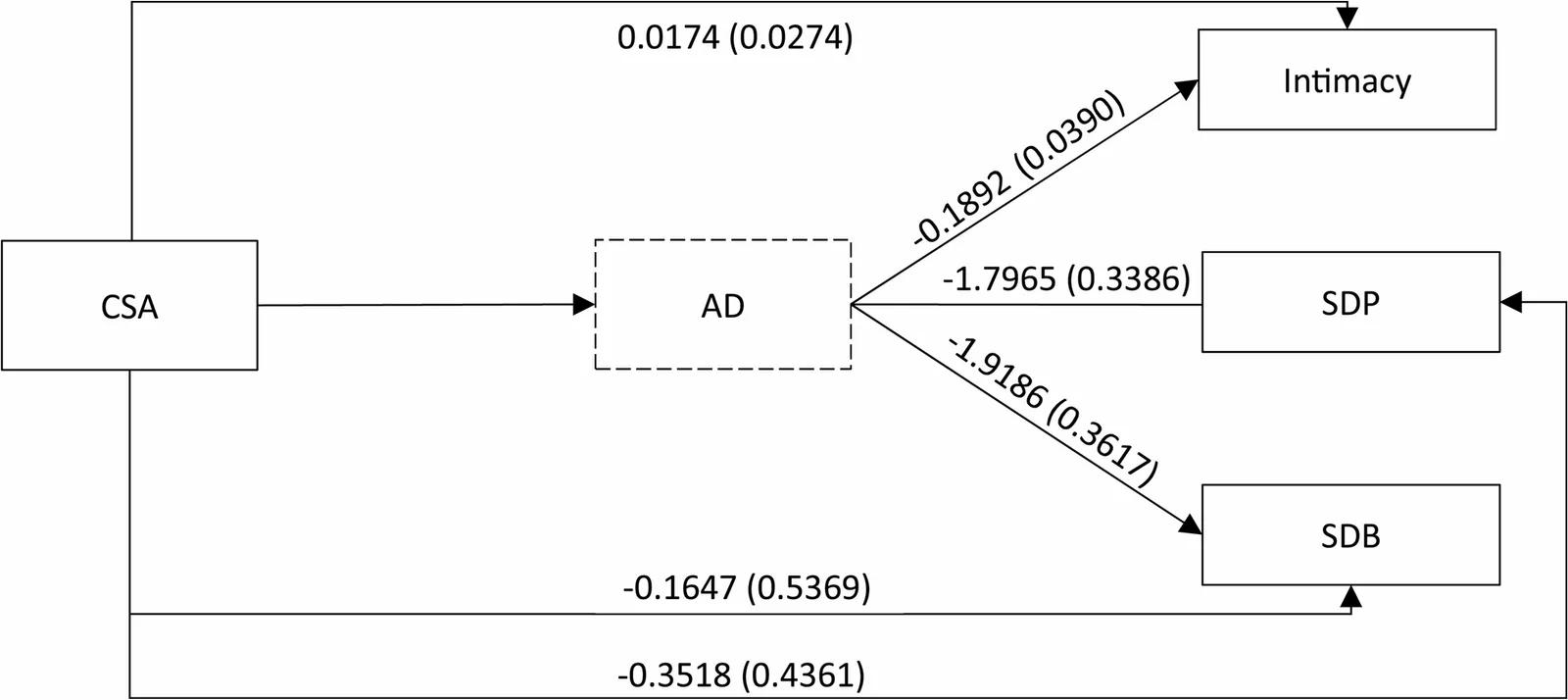
Total Direct and Indirect Effects on Intimacy Variable. **Note**. SDP: self-disclosure for personality; SDB: self-disclosure for body; AD: attachment dimensions. Paths from CSA to variables are the direct effects (c’); paths from CSA through AD are the indirect effects (c). **Statistical note**. Paths are b (SE) reported. Direct effect on Intimacy *p* = 0.5255 [−0.04, 0.07]; direct effect on SDP *p* = 0.4202 [−1.21, 0.51]; direct effect on SDB *p* = 0.7592 [−1.21, 0.51]; indirect effect on intimacy [−0.27, −0.11]; indirect effect on SDP [−2.49, −1.16]; indirect effect on SDB [−2.68, −1.25]

Finally, a series of multiple mediation analyses were conducted to examine the role of attachment dimensions in the relationship between CSA and the three intimacy variables, with gender included as a covariate in all models. Multiple mediation analyses revealed that CSA had no significant direct effect on intimacy or self-disclosure, both personal and bodily, once mediators were controlled for. However, a significant and negative overall indirect effect was found in all three models, indicating that abuse is associated with lower levels of intimacy and self-disclosure across attachment dimensions (Fig. 1).

**Fig. 2:**
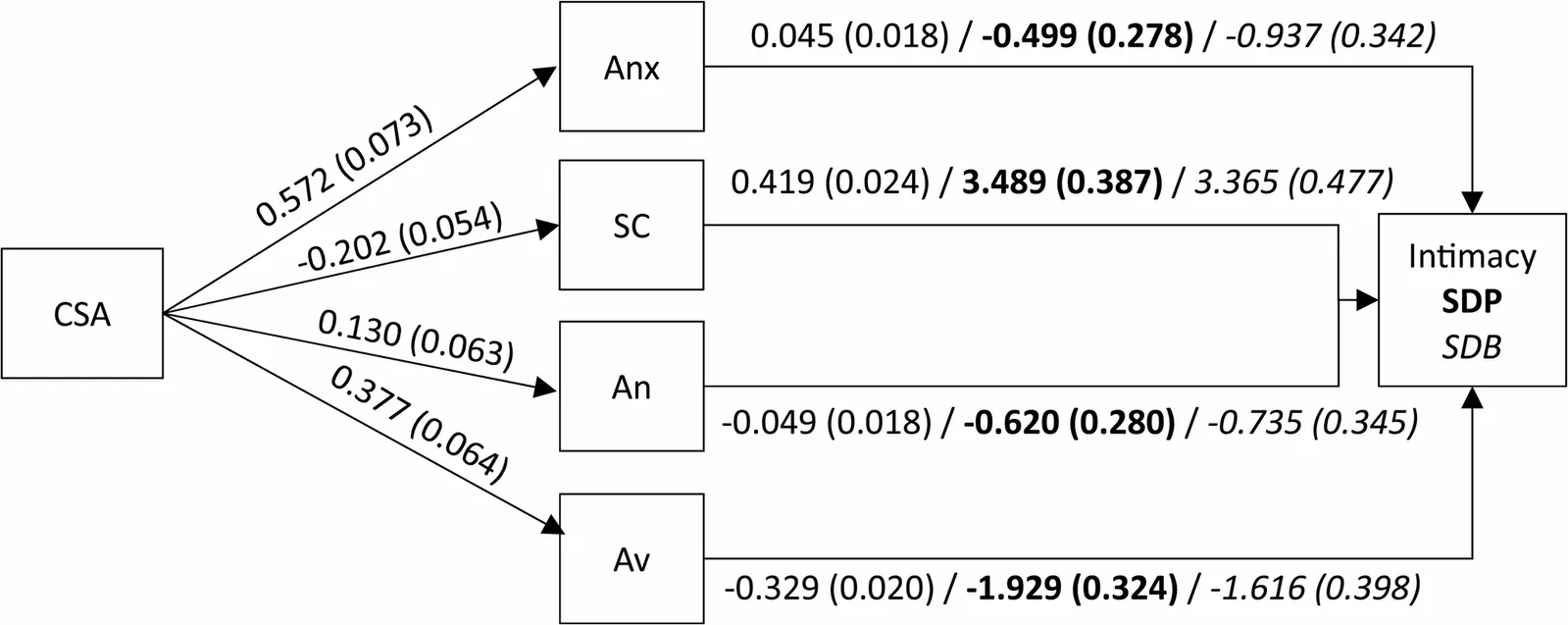
Partial Effects: Paths a and b. **Note**. *N* = 576. CSA values: 0 = No, 1 = Yes. Indirect effects are reported with bootstrapped 95% confidence intervals. Anx: Anxiety; SC: Socioemotional competence; An: Anger; Av: Avoidance; SDP: self-disclosure for personality; SDB: self-disclosure for body. Arrows from CSA are the ‘a’ paths and arrows from attachment dimensions are the ‘b’ paths. **Statistical note**. Paths are b (SE) reported. From CSA to (a paths): Anx. *p* < 0.001 [0.43, 0.72]; SC *p* < 0.001 [−0.31, −0.10]; An. *p* = 0.0406 [0.01, 0.25]; Av. *p* < 0.001 [0.25, 0.50]. From Anx. to: intimacy *p* = 0.0105 [0.01, 0.08]; SDP *p* = 0.0729 [−1.05, 0.05]; SDB *p* = 0.0064 [−1.61, −0.27]; from SC to: Intimacy *p* < 0.001 [0.37, 0.47]; SDP *p* < 0.001 [2.73, 4.25]; SDB *p* < 0.001 [2.43, 4.30]; from An. to: intimacy *p* = 0.0058 [−0.08, −0.01]; SDP *p* = 0.0273 [−1.17, −0.07]; SDB *p* = 0.0335 [−1.41, −0.06]; from Av. to: intimacy *p* < 0.001 [−0.37, −0.29]; SDP *p* < 0.001 [−2.57, −1.29]; SDB *p* = 0.0001 [−2.40, −0.83]

In terms of partial effects (Fig. 2; Table 6), Avoidance and Socioemotional Competence emerged as consistent mediators. CSA was associated with increased Avoidance and decreased Socioemotional Competence. In turn, greater Avoidance and lower Socioemotional Competence predicted a reduction in all three outcome variables. However, the role of attachment anxiety differed. For intimacy, this variable was a mediator where abuse (higher anxiety) was associated with an increase in intimacy. In contrast, for self-disclosure about the body, attachment anxiety was a mediator where abuse (higher anxiety) was associated with a decrease in bodily self-disclosure. It was not a significant mediator for self-disclosure about personality. Anger was not a significant mediator in any of the models, but it was for partial effects.

**Table 6:** Specific Indirect Effects of CSA on Intimacy variables

M variable	Y variable	b	SE	IC 95%
Avoidance	Intimacy	−0.1238	0.0244	[−0.18, −0.08]
	Self-disclosure for personality	−0.7269	0.1906	[−1.15, −0.40]
	Self-disclosure for body	−0.6089	0.1904	[−1.03, −0.28]
Anxiety	Intimacy	0.0258	0.0110	[0.01, 0.05]
	Self-disclosure for personality	−0.2855	0.1723	[−0.63, 0.04]
	Self-disclosure for body	−0.5356	0.2162	[−0.98, −0.14]
Socioemotional competence	Intimacy	−0.0847	0.0234	[−0.13, −0.04]
	Self-disclosure for personality	−0.7034	0.2035	[−1.13, −0.34]
	Self-disclosure for body	−0.6784	0.2101	[−1.13, −0.31]
Anger	Intimacy	−0.0063	0.0042	[−0.02, 0.00]
	Self-disclosure for personality	−0.0807	0.0617	[−0.23, 0.00]
	Self-disclosure for body	−0.0957	0.0755	[−0.27, 0.01]

*N* = 576. CSA values: 0 = No, 1 = Yes. Indirect effects are reported with bootstrapped 95% confidence intervals

Regarding the covariate Gender, a significant effect was only found on the attachment anxiety dimension, indicating that males reported significantly lower levels of attachment anxiety compared to females, although gender did not significantly predict any of the other attachment dimensions or outcome variables (Table 7).

**Table 7:** Gender as Covariable Effects on Mediators and Results Variables

Path	b	SE	*p*	IC 95%
Gender → Mediators (Covariable Paths)				
Gender → Avoidance	0.1041	0.0920	0.2585	[−0.08, 0.28]
Gender → Anxiety	−0.2280	0.1052	0.0307	[−0.43, −0.02]
Gender → Socioemotional competence	−0.1019	0.0770	0.1862	[−0.25, 0.05]
Gender → Anger	−0.0855	0.0909	0.3475	[−0.26, 0.09]
Covariable Effects (Gender to Y’)				
Gender → Intimacy	−0.0502	0.0376	0.1826	[−0.12, 0.02]
Gender → Self-disclosure for personality	−0.0965	0.5980	0.8719	[−1.27, 1.08]
Gender → Self-disclosure for body	0.2473	0.7362	0.7370	[−1.20, 1.69]

*N* = 576. Gender values: 1 = female, 2 = male. Indirect effects are reported with bootstrapped 95% confidence intervals

## Discussion

The findings of this study provide strong evidence of the persistent impact of CSA on relational and emotional capacities in adulthood, particularly on attachment styles and dimensions of intimacy. Overall, it confirms that individuals with a history of CSA have a more vulnerable attachment profile, manifested in insecure attachment styles and reduced levels of intimacy and self-disclosure.

First, regarding the gender variable, women accounted for 89.4% of those who reported having suffered CSA, showing a statistically significant association between gender and victimization. This finding is consistent with the literature, which points to a higher prevalence of CSA in women (Pan et al., 2020; Piolanti et al., 2025; Wang, 2024), both because of structural factors -such as unequal power in relationships and greater exposure to contexts of vulnerability- (Rechenberg & Schomerus, 2023), and a greater willingness to disclose these experiences (Okur et al., 2017; O’Gorman et al., 2024). From a gender perspective, this overrepresentation may also be influenced by differences in emotional socialization: women tend to be more familiar with the affective world and with the recognition of relational harm (Davis et al., 2020), whereas men, socialized under ideals of emotional self-reliance and control, may minimize or suppress the experience of trauma (Rechenberg & Schomerus, 2023), making it more difficult to identify and express. This difference is not only reflected in the prevalence of CSA, but also in subsequent affective patterns. In mediation analyses, gender showed a significant effect only on the anxiety dimension of attachment, where men reported significantly lower levels than women. However, gender did not present relevant effects on the other attachment dimensions or on intimacy variables, suggesting that, although it may modulate certain emotional aspects -such as fear of abandonment or the need for validation- (Melero & Cantero, 2024), the impact of CSA on attachment and intimacy in adulthood operates in a similar way in both genders. These findings invite us to consider gender not only as a demographic variable, but as a factor that cuts across both the experience and the expression of trauma.

Analysis by age group also yielded relevant data. Although no significant interactions were found between age and CSA on the intimacy variables, there was a main effect on the resolution of intimacy, with lower scores in people older than 24 who had experienced CSA. This could indicate that, over time, difficulties in building deep emotional bonds tend to consolidate if appropriate interventions are not received. Intimacy, if not resolved spontaneously, may crystallize into loneliness or isolation (Hoffman et al., 2020).

One of the most striking results is the significant difference in the dimensions of attachment anxiety and avoidance, with CSA victims showing higher levels in both dimensions. This indicates that these individuals more frequently experience fear of abandonment, doubts about their relational value and, simultaneously, a defensive need for emotional distancing (Melero & Cantero, 2024). This combination reflects an ambivalent and contradictory pattern, consistent with the fearful attachment style, which was most prevalent among the victims. In contrast, socioemotional competence, understood as the ability to adequately manage one’s own and others’ emotions in affective contexts, was significantly lower in the CSA group. This dimension is key to establish satisfactory and resilient relationships (Sagone et al., 2023), and its deficit suggests that the effects of trauma not only impact on the emotional sphere, but also complex interpersonal skills. Thus, from the cluster analysis, the distribution of attachment styles according to the experience of CSA reinforces these results. While 36.7% of people with no history of CSA were in the secure style, only 15.5% of CSA victims were in it. In contrast, 41.9% of victims adopted a fearful style, characterized by high emotional arousal without adequate resources for its management, suggesting that total emotional withdrawal is less frequent than ambivalence and over-arousal in this population (Årseth et al., 2009; Shen et al., 2021).

Although anger did not show significant differences between groups, it did act as a partial mediator in the relationship between CSA and the three dimensions of intimacy. This result suggests that, although its effect is more discrete, anger may interfere both in the adaptive resolution of the intimacy crisis and in emotional and bodily openness, probably as a defensive response to affective harm. Its role as a mediator suggests that certain expressions of hostility or irritability - possibly internalized or directed towards the intimate environment - are also part of the relational pattern affected by trauma (Camisasca et al., 2019; Coyle et al., 2014).

In terms of intimacy, significantly lower scores were also observed in the victim group, reaffirming the idea that trauma undermines the ability to generate deep and secure emotional bonds (MacIntosh & Ménard, 2021). This is reinforced by the results on the self-disclosure subscales on personality and body, where again lower levels are evident in people with a history of CSA. Bodily self-disclosure is a highly sensitive dimension in this population, given the invasive nature of the trauma, which may explain its differential effect (Petersson & Plantin, 2024).

A key and distinctive contribution of this study is the identification of specific attachment dimensions as the primary explanatory mechanisms linking CSA to adult intimacy. Our mediation analysis reveals that the impact of abuse is not linear, instead, it operates through internalized relational schemas —specifically avoidance and low socioemotional competence— which indirectly reduce adaptive crisis resolution and self-disclosure. This confirms that the consequences of abuse do not act in a linear fashion, but through internalized relational schemas that mediate how the person bonds with others (Helm et al., 2020; Riazi & Manouchehri, 2024). Another novel finding is the specific ambivalent pattern identified for attachment anxiety. This dimension presented a dual effect: acting as a positive mediator for general intimacy—likely driving a search for emotional closeness—while simultaneously serving as a negative mediator for both personality and bodily self-disclosure. This suggests that anxiety may, in certain cases, drive the search for emotional closeness (Clark et al., 2020; de Jager et al., 2021), albeit without the safety and comfort needed to include the body in this process of openness, or to show personality vulnerability (Manbeck et al., 2020). This discrepancy highlights a unique clinical nuance: for CSA survivors, the yearning for bond-seeking (driven by anxiety) may coexist with a profound lack of safety regarding physical and emotional vulnerability.

These findings have important implications for intervention and prevention. First, they reinforce the need for attachment-focused interventions that address not only the trauma narrative but also internalized relational patterns. Second, the dimension of self-disclosure needs to be carefully addressed, as it seems to be particularly affected in this population, probably reflecting a protective disconnection from vulnerability.

Finally, this study presents remarkable methodological and conceptual strengths that strengthen the validity of its findings. These include the size of the sample, which provided sufficient statistical power and allowed for robust multivariate analyses, as well as the use of validated instruments that accurately capture both attachment and intimacy dimensions. In addition, the design incorporated mediation analyses and control for covariates such as gender, which allowed for a better examination of the mechanisms underlying the relationship between CSA and attachment. However, certain limitations must also be considered. The use of a non-probability sample, together with the over-representation of women, limits the generalizability of the results to the general population. Also, contextual variables of CSA, factors that could modulate the impact of trauma on attachment and intimacy patterns, were not explored. Additionally, other forms of childhood maltreatment, such as physical abuse or neglect, which are often co-occurring with CSA and influence attachment patterns, were not assessed. Furthermore, the cross-sectional design and data collection through a single source, such as self-reporting, does not allow for clear causalities to be established, so inferences should be interpreted with caution. Retrospective self-reporting of CSA may involve some recall/attribution bias. The findings are understood as associations consistent with the model. Therefore, future research could benefit from longitudinal approaches to track the evolution of these bonds over time, as well as to assess the effectiveness of attachment and intimacy interventions in populations with a history of CSA.

## Data Availability

The datasets generated and/or analyzed during the current study have not been uploaded, as this was not considered necessary due to the data reported in the article are deemed sufficient to ensure the study’s replicability and the sensitive nature of it. The full dataset is available from the corresponding author upon reasonable request.
